# Household deprivation, comorbidities and COVID-19 hospitalization in 690,115 children/adolescents: Nico Dragano

**DOI:** 10.1093/eurpub/ckac129.673

**Published:** 2022-10-25

**Authors:** N Dragano, O Dortmann, J Timm, M Mohrmann, R Wehner, CJ Rupprecht, M Scheider, E Mayatepek, M Wahrendorf

**Affiliations:** 1 Institute of Medical Sociology, University Hospital Duesseldorf, Düsseldorf, Germany; 2 AOK Rhineland/Hamburg, Düsseldorf, Germany; 3 Institute of Virology, University Hospital Düsseldorf, Düsseldorf, Germany; 4 Department of General Pediatrics, Neonatology and Pediatric Cardiology, University Hospital Düsseldorf, Düsseldorf, Germany

## Abstract

**Background:**

Studies document that adults in disadvantaged socio-economic positions have elevated risks of a severe course of COVID-19, but it is unclear if this holds true for children. We investigate in this population-based study whether young people from socio-economically disadvantaged households in Germany had a higher risk of COVID-19 hospitalization compared with more affluent counterparts. We also examined if differences were related to comorbidities that predict severe courses in children.

**Methods:**

We included data from all 690,115 children and adolescents (0-18 years) enrolled in a statutory health insurance carrier. Daily hospital diagnoses of COVID-19 were recorded from 1.1.2020 to 13.7.2021. Logistic regressions were used to compare children from households with an indication of poverty (e.g. long- or short-term unemployed) with children from households with insurance holders in regular employment. We also assessed socio-economic characteristics of the area of residence. We controlled for age, sex, days under observation, nationality, and comorbidities (e.g. obesity).

**Findings:**

A COVID-19 hospital diagnosis was a rare event (n = 1637). Children of long-term unemployed parents had a 1·36 times (95% CI 1·21-1·51) higher adjusted odds of hospitalization compared with those of employed parents. Elevated odds were also found for short-term unemployed or low-wage employment. Those living in poor areas had a 3·02 (1·81-5·22) higher odds of hospitalization than those in less deprived areas. Comorbidities were strongly related to hospitalization, but their adjustment did not change main estimates for household deprivation.

**Discussion:**

Results suggest that children from poor households are at higher risk of severe courses of COVID-19 than their affluent counterparts. This underlies the need to implement effective Public Health strategies to protect deprived children from COVID-19 and other infectious disease even in high income countries such as Germany.

**Key messages:**

• Children and adolescents from poor families seem to be at higer risk for sever courses of COVID-19.

• Comorbidities were no key mediating factor in this study.

